# Retroperitoneoscopic robot-assisted laparoscopic partial nephrectomy during the second trimester of pregnancy: a case report and literature review

**DOI:** 10.1016/j.ijscr.2025.111483

**Published:** 2025-06-11

**Authors:** Yiman Zhang, Taoyue Zhao, Zhongyi Li, Xiao Jin, Zhaohui Wang, Haixiang Shen

**Affiliations:** a Department of Nursing, The Second Affiliated Hospital of Zhejiang University School of Medicine, China; b Department of Urology, The Second Affiliated Hospital of Zhejiang University School of Medicine, China; c Department of Obstetrics, The Second Affiliated Hospital of Zhejiang University School of Medicine, China

**Keywords:** Case report, Pregnancy, Renal cell carcinoma, Robot-assisted laparoscopic partial nephrectomy

## Abstract

**Introduction and importance:**

The incidental discovery of renal tumors during pregnancy necessitates a tailored treatment that carefully balances the health and well-being of both the mother and the developing fetus.

**Case presentation:**

The patient accidentally found a renal mass on abdominal ultrasound in the second trimester, and following MRI diagnosed cT1bN0M0 renal cancer. Following a multidisciplinary consultation and taking into account the patient's clinical status and preferences, retroperitoneoscopic robot-assisted laparoscopic partial nephrectomy (rRAPN) was successfully performed at 26 weeks of gestation. The procedure was completed without any postoperative complications and clear cell renal cell carcinoma was confirmed by histopathology. The postoperative recovery of the patient was uneventful and the baby was born safely at 38 weeks of gestation.

**Clinical discussion:**

Gestational renal tumor is usually detected by routine antenatal ultrasonography incidentally without any typical symptoms, of which 50 % are malignant. The clinical decision-making process poses significant challenges, necessitating meticulous risk-benefit analysis to balance tumor control efficacy with perinatal safety. The two principal surgical approaches for renal tumor resection are the transperitoneal and retroperitoneoscopic routes, with selection guided by tumor location, surgeon expertise, and patient-specific anatomical considerations.

**Conclusion:**

**r**RAPN is safe and effective in the second trimester. Personalized treatment should be made for each gestational RCC by a multidisciplinary team.

## Highlights

• The incidence of renal tumors during pregnancy is rare.
• A tailored treatment that carefully balances the health and well-being of both the mother and the developing fetus.
• Retroperitoneoscopic robot-assisted partial nephrectomy is safe and effective in the second trimester.

## 1 Introduction

Renal cell carcinoma (RCC) is a common malignancy of urinary system which accounts for 3 % of all cancer types, with 434,840 new cases worldwide estimated in 2022 [[1], [2], [3], [4]]. Urological malignancies during pregnancy are exceedingly rare (~13/1,000,000), while RCC accounting for the vast majority of reported cases [5,6]. Gestational RCC is usually detected by routine antenatal ultrasonography incidentally without any typical symptoms. However, because of the rarity, there is no evidence-based guidelines or consensus for the management of RCC during gestation currently. The clinical decision-making process poses significant challenges, necessitating meticulous risk-benefit analysis to balance tumor control efficacy with perinatal safety. Therefore, multidisciplinary consultation is crucial when making the individualized treatment.

Comparable to non-pregnant RCC, surgical intervention remains the main treatment for localized RCC in pregnancy. With surgical techniques advanced, minimally invasive laparoscopic surgeries, even robotic-assisted approaches, are performed for RCC rather than open surgeries [[7], [8], [9], [10]]. Currently, laparoscopic surgeries are commonly applied in patients during pregnancy, especially for patients scheduled with laparoscopic cholecystectomies [7,11]. Furthermore, the guidelines advocated by SAGES (Society of American Gastrointestinal and Endoscopic Surgeons) indicate that laparoscopic surgeries can be performed safely during pregnancy [12]. While for pregnant patients with metastatic RCC, systemic therapy combined with surgery and targeted drugs should be considered [13]. However, owing to the absence of evidence-based guidelines or consensus, the management of gestational RCC still remains controversial, particularly regarding the intervention timing and therapeutic strategy selection [5].

In this paper, we report our experience in the diagnosis and management of gestational RCC during the second trimester, detailing a case of a 26-week gestation patient with a right lower pole RCC that was successfully treated with retroperitoneoscopic robot-assisted partial nephrectomy (rRAPN). This case report has been reported in line with the SCARE checklist [14].

## 2 Case presentation

A 39-year-old woman, gravida 3 para 1, at 24 weeks of gestation, was referred to our hospital with a suspicious RCC which was located on the lower pole of right kidney. The patient was asymptomatic and was detected by routine antenatal ultrasonography. The patient delivered a healthy boy 17 years ago without any remarkable medical history. Physical examination revealed no abdominal tenderness or guarding, with no palpable masses detected upon systematic palpation, and the blood pressure was 113/78 mmHg. Baseline laboratory assessments, including complete blood count and comprehensive biochemical panel, demonstrated values within normal limits: hemoglobin 124 g/L (reference range: 113–151 g/L) and serum creatinine 47.4 μmol/L (reference range: 41.0–72.0 μmol/L).

**Fig. 1:**
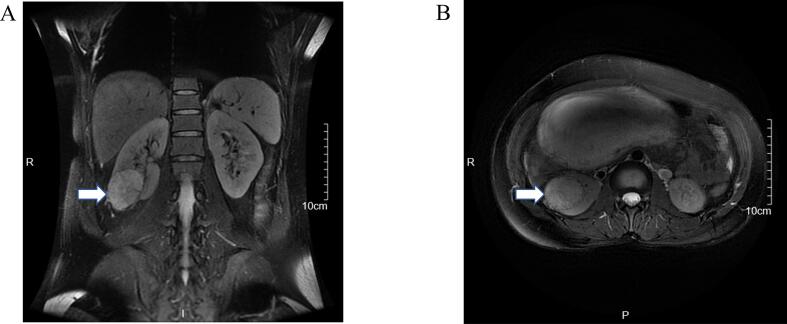
MRI demonstrated a 4.6 × 4.1 × 5.3 cm mass at the lower pole of the right kidney, exhibiting heterogeneous signal intensity on T2. Coronal (A) and Axial (B). The white arrow indicates the tumor.

**Supplementary Fig. 1:**
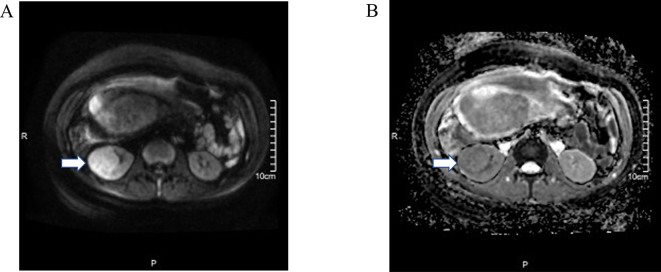
MRI demonstrated that the renal mass showed restricted diffusion, with DWI (A) showing hyperintensity and the ADC (B) revealing decreased values.

The following MRI demonstrated a 4.6 × 4.1 × 5.3 cm mass at the lower pole of the right kidney, exhibiting heterogeneous signal intensity on T2, restricted diffusion (DWI hyperintensity with reduced ADC values), radiologically suspicious for renal cell carcinoma without any enlarged lymph nodes or distant metastases (cT1bN0M0) (Fig. 1 and Supplementary Fig. 1). With regard to the local disease and suspicious renal carcinoma, the multidisciplinary consultation including urology, anesthesiology, neonatology and obstetrics and the patient's wish were considered, rRAPN was scheduled for the patient.

**Fig. 2:**
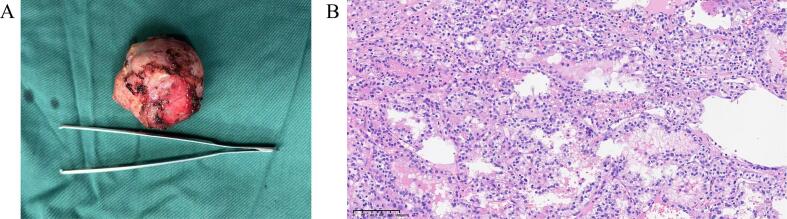
Gross specimen of the tumor (A) and HE staining of the tumor (B).

**Supplementary Fig. 2:**
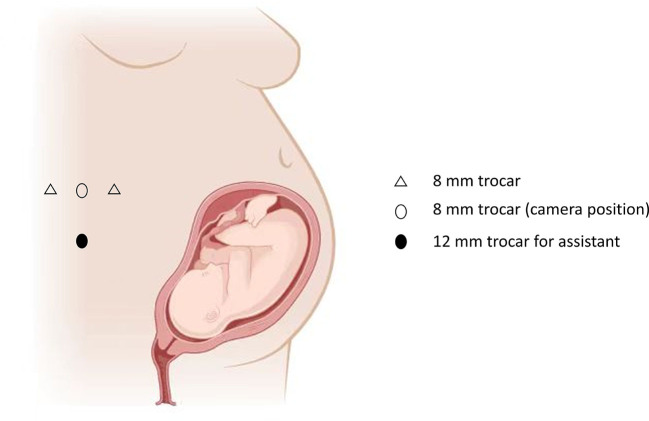
The figure showed the robotic trocar positions.

In order to avoid the interference with uterus, the rRAPN was performed with the da Vinci robot system Xi (Intuitive Surgical, Sunnyvale, CA, USA) under general anesthesia. During the surgical procedure, the patient was in left recumbent position. Through blunt dissection and balloon dilation with a small incision 2 cm above the iliac crest of the midaxillary, the retroperitoneal space was established, followed by the insertion of the trocar for the camera. The other two trocars were inserted at the level of the costal arch on the anterior axillary and below the posterior axillary twelfth rib on the posterior axillary, respectively (Supplementary Fig. 2). After identification of the renal artery and vein and the mass on the lower pole of the right kidney with removing the perirenal tissue, the renal artery was clamped. Then, the mass was dissected by monopolar scissors completely. Subsequently, a continuous renorrhaphy was performed, followed by the removal of the bulldog clamps, and the surgery was concluded with the excision of the tumor. The total surgical time was 80 min with warm ischemia time of 15 min and the estimated blood loss was 100 mL. During the whole procedure, the fetal heart beat sounds were monitored with obstetrics standby and the pressure of pneumoperitoneum was strictly maintained <12 mmHg. The final pathology analysis confirmed the diagnosis of clear cell renal cell carcinoma with negative surgical margins (Fig. 2).

The recovery of the patient was uneventful and she was discharged on the eighth postoperative day. Subsequently, the patient delivered a healthy baby at 38 weeks gestation by cesarean section. The follow-up of one and a half years, the patient and her baby were both in good condition.

## 3 Discussion

The incidence of cancer diagnosis during pregnancy is rare, with about 0.1 % of pregnant women diagnosed with cancer before delivery [13,15]. RCC diagnosed during pregnancy is extremely rare, with only approximately 100 cases having been reported in the literature [16]. We report the case diagnosed with RCC at 24 weeks gestation and underwent the surgery of rRAPN.

As reported, diagnosis of cancer during pregnancy is rare, and RCC diagnosed in pregnant woman is extremely rare which was firstly reported in 1956 [17]. As far as we know, there are only about 100 cases of gestational RCC reported in the literature [16]. With regard to the etiology, obesity, smoking and hypertension are identified as risk factors for RCC [18]. Furthermore, it is reported that the hormones (estrogen and progesterone) during pregnancy and the number of pregnancies are associated with the development of gestational RCC [19,20]. However, the underlying mechanisms are not clarified yet.

Comparable to non-pregnant RCC, gestational RCC are usually asymptomatic. According to the reports in the literature, the main symptoms are pain, hematuria and hypertension, which are also related to pregnancy-associated disorders [21]. Therefore, the early diagnosis of gestational RCC is full of challenges.

Because of lacking the typical symptoms, the gestational RCC are usually discovered by routine antenatal ultrasonography accidently. Considering of the potential teratogenic risks for the fetus, CT scan is not suggested during pregnancy [22]. With no adverse maternal or perinatal affects, ultrasonography is the preferred examination method for routine antenatal evaluation, and suspicious RCC [23]. For diagnosis of small renal masses (<3 cm) and further evaluation of the tumor size, local invasiveness, lymph node involvement and tumor thrombus, MRI is suggested with its good sensitivity and accuracy [24]. While due to the potential risk of stillbirth and neonatal death, contrast MRI is not suggested [25].

As for the management of RCC during pregnancy, the multidisciplinary consultation and the wish of the patient should be taken into consideration when decision making [15,26]. With localized RCC, surgical intervention may be recommended. However, the individualized schedule of surgical timing and approach should be made, with the stage of the tumor and the trimester of the pregnancy considered. For RCC diagnosed in the first trimester of pregnancy, it is recognized that surgery should be performed without delay, regardless of the risks of spontaneous abortion or congenital abnormalities [27]. Also, termination of pregnancy may be an option for RCC diagnosed early in the gestation prior to surgery [5]. When it comes to the third trimester, surgical removal of the renal mass and a cesarean section can be applied simultaneously [28]. If the RCC is detected when the estimated due date approaching, spontaneous vaginal delivery followed by the surgery of RCC is an option [16,29,30]. However, for the RCC during the second trimester, the management remains controversy. Some scholars advocate postponing the surgery until 28 weeks of gestation, allowing the fetal lung to develop sufficiently for safe delivery [31]. While the others indicate that the second trimester is the appropriate time for surgery, because of the increasing risk of preterm labor and limited operative space with gestation progressing. Although the estimated doubling time of RCC is about 300–500 days, it was reported that the risk of metastasis increased by 25 % for every additional centimeter of renal mass maximum diameter [32,33]. Also, rapidly enlarging of renal tumor in pregnancy was reported in the literature, which could result in poor prognosis [[34], [35], [36]]. That is, the resectable tumor would progress into a palliative situation for the delay of surgery. Therefore, immediate surgical intervention regardless of pregnancy trimester is an alternative option for some cases.

The two principal surgical approaches for renal tumor resection are the transperitoneal and retroperitoneoscopic routes, with selection guided by tumor location, surgeon expertise, and patient-specific anatomical considerations. While the transperitoneal approach provides a generous working space advantageous for complex tumor dissection, it offers suboptimal exposure of the retrorenal anatomy (particularly the renal hilum and posterior parenchyma) and necessitates extensive uterine retraction in female patients, which may elevate preterm labor risks during pregnancy. The retroperitoneoscopic approach, despite constrained by a limited working space, provides direct access to the renal hilum and retroperitoneal vasculature without requiring visceral mobilization. This technique minimizes uterine manipulation due to its extraperitoneal trajectory, thereby reducing intraoperative hemodynamic fluctuations and preserving uteroplacental perfusion in gravid patients.

Consistent with the prior cases published in the literature [37], RCC of this patient was found by routine antenatal ultrasonography accidently without the typical clinical manifestations. Given the large size and the suspicious RCC, this case in our hospital was scheduled for partial nephrectomy with multidisciplinary consultation. To minimize the uterine interference and achieve early control of the renal artery, rRAPN was performed. As reported in the literature, there are only 7 documented cases of gestational RCC treated with robotic surgery [[7], [8], [9], [10],[38], [39], [40]], comprising six partial nephrectomies and one radical nephrectomy. Similar to the case reported by Völler et al., the retroperitoneoscopic approach was implemented within a confined anatomical space, optimally leveraging the technical advantages of the da Vinci robotic system [41]. The operative time was 80 min with total ischemia time of 15 min, which was consistent with the outcomes reported by Völler et al. (95 min of total operative duration with 15 min of ischemia time). To the best of our knowledge, this is the second report of renal cancer during pregnancy treated with rRAPN successfully.

RCC with inferior vena cava thrombus diagnosed during pregnancy is extremely rare, which has been reported with two cases [42,43]. The two cases were at 24 and 25 weeks of gestation respectively, with tumor stage of cT3bN0M0. Both of them were scheduled for delivery of the baby by cesarean section followed by radical nephrectomy with concomitant IVC thrombectomy. Molecular targeted therapy was arranged subsequently. For metastatic RCC in pregnancy, a recent case treated with ipilimumab and nivolumab, demonstrated favorable maternal and fetal outcomes alongside tumor response [44]. However, the data on the use of molecular targeted therapy during pregnancy are limited.

Due to the rarity, we have encountered only one case of RCC during pregnancy which was treated with rRAPN. Our successful experience suggests that this approach may serve as a reliable option for gestational RCC. However, due to the limited number of reported cases, further studies are required to validate its efficacy and safety.

## 4 Conclusion

The diagnosis of RCC in pregnancy is rare. Ultrasonography and MRI are preferable examinations for RCC screening and diagnosis in pregnancy. The individualized treatment should be made with the multidisciplinary consultation and the wish of the patient. rRAPN emerges as a safe, feasible, and effective treatment option for managing localized RCC during pregnancy.

The following are the supplementary data related to this article.

Supplementary Table 1 Timeline of the clinical course. Supplementary Table 1

## Author contribution

Yiman Zhang and Taoyue Zhao wrote the manuscript. Ningping Chu and Zhaohui Wang were responsible for patient follow-up and collection of relevant clinical data. Zhongyi Li, Xiao Jin and Haixiang Shen reviewed the manuscript.

## Consent for publication

Written informed consent was obtained from the participant for publication of identifying information/images.

## Ethical approval

Not applicable for this case report.

## Guarantor

All authors accept full responsibility for the case report.

## Research registration number

Not applicable.

## SCARE guideline

The work has been reported in line with the SCARE criteria [14].

## Funding

This case report was not supported by any funding.

## Conflict of interest statement

The authors declare no competing interests.

## Data Availability

No datasets were generated or analysed during the current study.
