# Supplementary material for: Retroperitoneoscopic robot-assisted laparoscopic partial nephrectomy during the second trimester of pregnancy: a case report and literature review
Source: Int J Surg Case Rep. 2025 Jun 11;133:111483. doi: 10.1016/j.ijscr.2025.111483 (PMC12212111; doi:10.1016/j.ijscr.2025.111483)
Supplement: Supplementary Table 1 — Timeline of the clinical course. [file mmc3.docx]

Table: Timeline of the clinical course.

| Episode | Situation |
| --- | --- |
| 24th of pregnancy | A renal mass was found |
| two weeks after diagnosis | rRAPN was performed |
| 8th postoperative day | The patient was discharged |
| one and a half months after surgery | A healthy baby was delivered |
| every 3 months afterwards | Routine follow-up revealed no abnormalities |
| 17th postoperative month | The patient and the baby were both in good condition |
